# Minoxidil May be a Partial Agonist of Estrogen Receptor Alpha

**DOI:** 10.17912/micropub.biology.002173

**Published:** 2026-06-02

**Authors:** Reem Elzaghir, Kailah C. Collins, Alaa Abdulshafi, Mostafa Ismail, Nora Asleh, Maya Saeed, Diego E. Galarza-Ramirez, Eduardo Valladares, Ashlee Lewis, Samantha Mena, Antonina Pizzo, Antonela Shyti, Nicole A. Najor, Marwa K. Abdel Latif, Mara R. Livezey

**Affiliations:** 1 Department of Chemistry & Biochemistry, University of Detroit Mercy, Detroit, MI, US; 2 Department of Pharmaceutical Science, Wayne State University, Detroit, MI, US; 3 Department of Biology, University of Detroit Mercy, Detroit, MI, US

## Abstract

Minoxidil (MX) is a common treatment for androgenetic alopecia (AGA). While there are many proposed mechanisms through which MX may increase hair growth, a clear connection to sex-hormone pathways has yet to be established. With recent evidence suggesting that MX may directly bind to Androgen Receptor (AR) and act as an anti-androgen, we investigated whether MX might also exhibit estrogenic activity. Estrogen-dependent cell lines, tryptophan emission, and computational docking were used to probe the possible Estrogen Receptor α (ERα) agonist activity of MX. Preliminary results suggest MX may be a partial agonist of ERα.

**Figure 1. Minoxidil (MX) stimulates estrogen-dependent cell growth by binding and activating Estrogen Receptor α (ERα):** A) Line-stick structures of 17β-estradiol (E _2_ ), minoxidil (MX), and minoxidil intermediate (MXI). T47D and MCF-7 cell proliferation after 4 days of treatment with B) EtOH, E _2_ , or MX; C) EtOH, E _2_ , or MXI; D) EtOH, E _2_ , 100 pM E _2_ and 4-hydroxytamoxifen (4-OHT), or 1-20 nM MX and 1 µM 4-OHT. E) Tryptophan emission spectrum of 200 nM full-length ERα with E _2_ or MX. F) Representative images of E _2_ (left) and MX (right) docked to the ERα ligand-binding domain (PDB ID: 1ERE). Key residues are labeled with some interaction distances shown. G) Average docking energies of E _2_ (n=53) and MX (n=66) bound to ERα. Data in B-D and G is average ± standard deviation; with at least n=3 biological replicates for B-D. All statistical analyses are Student’s t-test where n.s.=not significant; *p<0.05, **p<0.01, ***p<0.001, and ****p<0.0001.

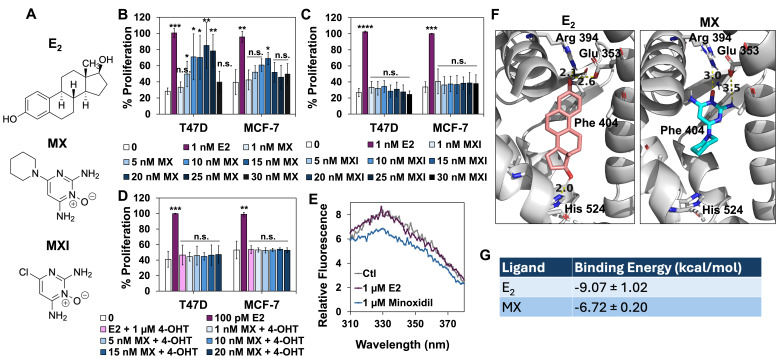



## Description

For nearly 40 years, Minoxidil (MX) has been used to treat androgenetic alopecia (AGA), a condition accounting for 95% of all hair loss.(Shen et al., 2023) There are many proposed mechanisms by which MX is thought to increase hair growth, including activation of voltage-gated K ^+^ channels, reducing inflammation, and increasing angiogenesis.(Abdul et al., 2003; Han et al., 2004; Shen et al., 2023; Shorter et al., 2008; Uno et al., 1987; Yum et al., 2017) While AGA is thought to be influenced by levels of sex hormones including testosterone and estradiol (E _2_ ), (Alonso & Rosenfield, 2003; Desai et al., 2024; Freites-Martinez et al., 2018) until recently, MX was not known to directly impact the human endocrine system.

MX is associated with hormone levels and endocrine disorders, specifically androgens and hyperandrogenism, and has also been shown to impact menstrual cycles.(Liu et al., 2025; Vexiau et al., 2002; K. N. Williams et al., 2024) Three recent studies suggest that MX may exert more direct anti-androgenic effects.(Gupta et al., 2023; Hsu et al., 2014; Shen et al., 2023) MX binds to Androgen Receptor (AR) at a surface pocket, inhibiting transactivation and expression of AR-regulated genes in AR-positive prostate cancer and dermal papilla cells, independent of its K ^+^ channel activity.(Hsu et al., 2014) Furthermore, MX has been shown to downregulate enzymes responsible for androgen synthesis in dermal papilla cells, including cytochrome P450 17A1, which acts early in sex-hormone synthesis and 5α-reductase 2, the enzyme responsible for converting testosterone to dihydrotestosterone.(Gupta et al., 2023; Shen et al., 2023) Only modest evidence suggests that MX may also impact estrogen-regulated pathways. Molecular docking demonstrated possible binding of MX to aromatase, the enzyme that converts testosterone to E _2_ , and treatment of dermal papilla cells with MX led to increased E _2_ synthesis.(Shen et al., 2023)

Given the limited existing evidence, known cross-reactivity of E _2_ and androgens for AR and Estrogen Receptor α (ERα),(Gao et al., 2005; Kuiper et al., 1997; Rider et al., 2009; Veldscholte et al., 1992; Yamazaki & Reddy, 2025; Yeh et al., 1998) and similarity of AR and ERα ligand-binding domain structures, we hypothesized that if MX binds to AR, it might also bind to ERα. Therefore, in addition to being anti-androgenic, MX may also be estrogenic. Here, we leverage T47D and MCF-7 breast cancer cell lines that express wild-type ERα and are estrogen-dependent for proliferation to ascertain whether MX may bind to and activate ERα.

MX caused a significant and dose-dependent increase in proliferation for both T47D and MCF-7 cells up until 15-20 nM ( Figure 1A, B). An MX concentration 20 times greater than E _2_ reached 80% of the maximum effect in T47D cells, suggesting MX may be a weak ERα agonist. Interestingly, there is a decrease in proliferation from 20-30 nM MX in both cell lines. While not shown here, we observed a “double hump” effect of MX on proliferation in T47D cells when range-finding the dose curve. This suggests that MX may have toxicity at some intermediate doses and more than one molecular target within cells at higher nM-µM concentrations. To understand if the effect of MX is structure-dependent, we used a minoxidil intermediate 6-chloropyrimidine-2,4-diamino-3-oxide (MXI) that lacks the piperidine ring ( Figure 1A ). Supporting a structure-dependent effect of MX on cell proliferation, MXI did not promote cell growth in either T47D or MCF-7 cells ( Figure 1C ). Given the ability of MX to promote proliferation in E _2_ -dependent cell lines, we asked whether the competitive inhibitor of E _2_ and selective Estrogen Receptor modulator, 4-hydroxytamoxifen (4-OHT) would inhibit MX-induced cell growth.(Fanning et al., 2016; Katzenellenbogen et al., 1984; Shiau et al., 1998) We saw complete inhibition of MX-induced proliferation when cells were co-treated with 4-OHT ( Figure 1D ). This further suggests a possible ERα agonist action of MX, and given 4-OHT is a competitive inhibitor, indicates that MX may directly bind to ERα.

We performed a tryptophan emission assay using full-length ERα to determine if MX might bind ERα. There was a decrease in fluorescence observed at 330 nm upon addition of MX ( Figure 1E ). The decrease may indicate quenching of tryptophan emission due to conformational and environmental change, suggesting that MX may bind to ERα.(Ghisaidoobe & Chung, 2014; Lakowicz, 2006; Nair et al., 2005) To probe whether MX’s pro-proliferative effect may be from binding to the ERα ligand-binding site, we used the platform SwissDock with computation through AutoDock Vina to dock E _2_ or MX to the ERα ligand-binding domain (PDB ID: 1ERE; ERα bound to E _2_ ).(Brzozowski et al., 1997) E _2_ docking in the ligand-binding site showed binding orientation and interactions similar to the original crystal structure, with H-bonding interactions to Glu 353 (2.6 Å), Arg 394 (2.1 Å), and His 524 (2.0 Å), pi-pi stacking with Phe 404, and nonpolar contacts including Ala 350, Leu 387, Ile 424, and Leu 525 ( Figure 1F, left). MX was also found docked within the ligand-binding site. Given MX’s shorter molecular length and asymmetrical polarity ( Figure 1A ), we only saw H-bonding on one end of the binding site in any one pose, either to Glu 353 (3.5 Å) and Arg 394 (3.0 Å) or to His 524 (2.2 Å, not shown) ( Figure 1F, right). In all cases, H-bonding distances for MX were longer than with E _2_ . pi-pi stacking with Phe 404 was common, but fewer overall nonpolar contacts were made between MX and ERα compared to E _2_ . While docking is an imperfect measure of binding affinity, E _2_ bound tightly in our simulations ( Figure 1G ). Additionally, MX docking showed reduced H-bonding and nonpolar interactions, which likely contribute to weaker binding of MX to the ERα ligand-binding site ( Figure 1G ) and support its weaker pro-proliferative action in cells.

Since MX is sufficient to promote proliferation of E _2_ -dependent cell lines and proliferation is inhibited by 4-OHT ( Figure 1B, D), we propose that MX may be a partial agonist of ERα. Previous studies on AR and in MCF-7 cells used 100 nM - 100 µM MX; studies of its K ^+^ channel agonism, ability to induce VEGF, etc. use 1 mM MX.(Abdul et al., 2003; Hsu et al., 2014; Shen et al., 2023; Shorter et al., 2008; Yum et al., 2017) We therefore suggest the pro-proliferative effect of MX that we see in T47D and MCF-7 cells at low nM concentrations further supports specific action through ERα. However, the exact mechanism through which MX binds to ERα, and the extent of agonist activity remains unknown. Aligning with MX’s partial agonist activity, our tryptophan emission spectra suggest that MX may induce a conformational change in ERα distinct from the one induced by E _2_ , given their substantially different emission traces ( Figure 1E ). However, our ability to interpret this result is limited without more robust data from x-ray crystallography or cryo-EM. A previous crystal structure of MX binding to a surface pocket on AR combined with our tryptophan emission data suggests it is also possible that MX does not bind in the ligand-binding site of ERα as was modeled in our computational simulations ( Figure 1F, G). Therefore, studies demonstrating MX-induced nuclear ERα-ERE (estrogen response element) recruitment and modulation of ERα-responsive genes such as GREB1, Progesterone Receptor, and IL1-R1 will be critical to confirming if the agonist activity we are seeing here is through direct binding and activation of ERα by MX. Partial agonism of ERα by MX may describe a novel action of this treatment for androgenetic alopecia.

## Methods

*Reagents*

Minoxidil (MX), 17β-estradiol (E _2_ ), (Z) 4-hydroxytamoxifen (4-OHT), resazurin sodium salt (Alamar Blue), Minimum Essential Medium (MEM), RPMI-1640, and Fetal Bovine Serum (FBS) were purchased from Sigma Aldrich. Additional FBS was obtained from VWR and charcoal-dextran stripped (CD-FBS) for hormone-free assays. Full-length ERα was purchased from Active Motif.

*Synthesis of 6-chloropyrimidine-2,4-diamino-3-oxide*

The N-oxide intermediate (MXI) was adapted from patented processes (EP0295218B1 and US4866174A).(Botre’, 1992; Lamsa, 1989) 0.25 g of 2,6-diamino-4-chloro-pyrimidine was dissolved in 10 mL of methanol and heated to 30–40 °C until dissolved. 0.53 g of magnesium monoperoxiphthalate (MMPP) was incrementally added over 20 minutes (2:1 mole ratio), then the reaction was refluxed at 40 °C for 2 hours. The formation of the N-oxide intermediate was confirmed with Rf = 0.4 using silica oxide thin layer chromatography (TLC) and a solvent mixture of 5:4:1 of CH _3_ Cl/MeOH/AcOEt. The white milky solution was vacuum filtered and washed with 10 mL of cold methanol and recrystallized twice using 15 mL of DI water. The creamy white crystals were filtered and washed with cold DI water and methanol. The solid was left to dry in a vacuum oven at 30 °C overnight. The solid melting point was confirmed at 187 – 189 °C and GC-MS base peak of 145.3 m/z with retention time (RT) = 10.054 minutes.

*Cell Maintenance*

T47D cells were courtesy of David J. Shapiro and MCF-7 cells were from ATCC. T47D and MCF-7 cells were grown in MEM with either 10% or 5% FBS, respectively. Cells were used until passage 30.

*Cell Proliferation Assay*

T47D or MCF-7 cells were split into MEM containing 10% or 5% CD-FBS, respectively. Two days later, cells were plated at 1,000 (MCF-7) or 2,000 (T47D) cells/well in a 96-well plate, in MEM with CD-FBS. The next day, vehicle control (EtOH), E _2_ , 4-OHT, MX, or MXI were added. Cells were allowed to proliferate for 4 days, changing media halfway through and measured with Alamar Blue on the fourth day on a Synergy LX Fluorescent Plate Reader with excitation at 530 nm and emission at 590 nm.

*Tryptophan Emission*

Full-length ERα was diluted to 200 nM in Tris-Buffer (50 mM Tris/HCl pH 8.0, 150 mM KCl, 2 mM DTT, 1 mM EDTA, and 10% glycerol).(Nair et al., 2005) 1 µM E _2_ or MX was added and incubated for 10 minutes at 37 °C. Spectra were read on a Varian Cary Eclipse Fluorescence Spectrometer using a Quartz cuvette with excitation at 295 nm and emission from 310-380 nm and 5 nm emission slits.

*Molecular Docking*

PDB ID: 1ERE was edited to contain only the A chain without ligand and hydrogens were added in the MolProbity Web Tool.(Brzozowski et al., 1997; C. J. Williams et al., 2018) Simulations were performed on the SwissDock platform, with calculations from AutoDock Vina (1.2.0).(Bugnon et al., 2024; Eberhardt et al., 2021; Grosdidier et al., 2011; Trott & Olson, 2010) 1ERE was docked with either E _2_ or MX using the following parameters: ligand structures were obtained from PubChem in SMILES format, coordinates for docking were centered on the ligand-binding site (9.366,  47.674, 130.339), search box size was 18 Å x 18 Å x 18 Å, and sampling exhaustivity was 64 for maximum computational effort. Results were visualized and interaction distances measured in PyMOL (version 3.1.8) and dockings were verified to be in the ligand-binding site before inclusion in the dataset.( *The PyMOL Molecular Graphics System, Version 2.0 Schrödinger, LLC.* , n.d.)
